# Mutation of the EPHA2 Tyrosine-Kinase Domain Dysregulates Cell Pattern Formation and Cytoskeletal Gene Expression in the Lens

**DOI:** 10.3390/cells10102606

**Published:** 2021-09-30

**Authors:** Yuefang Zhou, Thomas M. Bennett, Philip A. Ruzycki, Alan Shiels

**Affiliations:** Department of Ophthalmology and Visual Sciences, Washington University School of Medicine, St. Louis, MO 63110, USA; yuefangzhou@wustl.edu (Y.Z.); bennetttm@wustl.edu (T.M.B.); p.ruzycki@wustl.edu (P.A.R.)

**Keywords:** lens, ephrin receptor, cell patterning, cytoskeleton, cataract

## Abstract

Genetic variations in ephrin type-A receptor 2 (EPHA2) have been associated with inherited and age-related forms of cataract in humans. Here, we have characterized the eye lens phenotype and transcript profile of germline *Epha2* knock-in mutant mice homozygous for either a missense variant associated with age-related cataract in humans (*Epha2*-Q722) or a novel insertion-deletion mutation (*Epha2*-indel722) that were both located within the tyrosine-kinase domain of EPHA2. Confocal imaging of ex vivo lenses from *Epha2*-indel722 mice on a fluorescent reporter background revealed misalignment of epithelial-to-fiber cell meridional-rows at the lens equator and severe disturbance of Y-suture formation at the lens poles, whereas *Epha2*-Q722 lenses displayed mild disturbance of posterior sutures. Immunofluorescent labeling showed that EPHA2 was localized to radial columns of hexagonal fiber cell membranes in *Epha2*-Q722 lenses, whereas *Epha2*-indel722 lenses displayed disorganized radial cell columns and cytoplasmic retention of EPHA2. Immunoprecipitation/blotting studies indicated that EPHA2 formed strong complexes with Src kinase and was mostly serine phosphorylated in the lens. RNA sequencing analysis revealed differential expression of several cytoskeleton-associated genes in *Epha2*-mutant and *Epha2*-null lenses including shared downregulation of *Lgsn* and *Clic5*. Collectively, our data suggest that mutations within the tyrosine-kinase domain of EPHA2 result in lens cell patterning defects and dysregulated expression of several cytoskeleton-associated proteins.

## 1. Introduction

First identified as epithelial cell kinase (eck), ephrin type-A receptor 2 (EPHA2) belongs to the largest subfamily of receptor tyrosine kinases that were originally discovered in a human erythropoietin-producing-hepatoma (EPH) cell line [1,2]. EPH receptors and their membrane-bound EPH receptor interacting ligands, or ephrins, play key signaling roles in embryonic development including tissue patterning, neurogenesis and vasculogenesis, adult tissue physiology including bone homeostasis and insulin secretion along with various diseases including cancers and neurodegeneration [3,4,5]. The mammalian EPH/ephrin receptor subfamily comprises 14 receptors divided into type-A (EPHA1-8, 10) and type-B (EPHB1-5) that preferentially interact with ephrin type-A (EFNA1-5) and type-B (EFNB1-3) ligands, respectively, to elicit forward (receptor-driven) or reverse (ligand-driven) bidirectional signaling in neighboring cells. Like other receptor tyrosine kinases, EPHA2 shares a type-1 (single-pass) transmembrane glycoprotein topology with several functional domains including an extracellular (N-terminal) ligand binding domain and an intracellular (C-terminal) tyrosine kinase (TK) signaling domain and a sterile-alpha-motif (SAM) domain implicated in modulating kinase activity and receptor dimerization [6,7]. Canonical forward signaling by EFNA1-EPHA2 often promotes cell–cell repulsion accompanied by EPHA2 oligomerization, phosphorylation, and kinase activation, whereas EPHA2-EFNA1 reverse signaling elicits kinase-independent cell–cell adhesion or repulsion depending on the specific cellular–extracellular context [8,9]. In addition, EPHA2 possesses ligand-independent kinase activity in many cultured tumor cell types [8,10] and overexpression of EPHA2 serves both as a prognostic marker and therapeutic target in various human epithelial cancers (e.g., breast, gastric, and lung), glioblastoma, and melanoma, whereas EPHA2 sequence variants have been associated with susceptibility to Kaposi’s sarcoma [9,11,12]. In addition, EPHA2 serves as a receptor for the growth factor progranulin [13] and several infectious agents including oncogenic viruses and fungal pathogens, and is involved in blood–brain barrier breakdown during malarial infection [14,15,16].

Besides cancer and infectious diseases, EPHA2 has been repeatedly linked with clouding of the eye lens or cataract(s)—a leading cause of visual impairment worldwide [17]. Currently, at least 23 coding, mutations in the human EPHA2 gene (*EPHA2*) underlie inherited, mostly autosomal dominant, forms of early-onset cataract often with a variable clinical morphology described as nuclear, cortical, and posterior polar/sub-capsular opacities depending on their location within the lens [18] (https://cat–map.wustl.edu/; accessed on 30 July 2021). Most *EPHA2* mutations underlying inherited cataract are missense or frameshift with the majority located in cytoplasmic regions of the receptor including the SAM and TK domains. In addition to relatively rare forms of inherited cataract, at least 12 common single nucleotide variants in *EPHA2* (mostly non-coding) including one non-synonymous coding variant (p.R721Q) located in the TK domain have been associated with susceptibility to the much more prevalent forms of age-related nuclear, cortical, and posterior sub-capsular cataracts [19,20] (https://cat–map.wustl.edu/; accessed on 30 July 2021). Further, in addition to such germline cataract-risk variants, *EPHA2* coding variants predicted to be functionally deleterious have been found in genomic DNA from lenses of adults over 50 years of age raising the possibility that somatic *EPHA2* variants may also contribute to the risk for age-related cataract [21].

The crystalline lens is a transparent, ellipsoidal, biomechanical structure that plays a critical role in anterior eye development and variable fine-focusing of images onto the photosensitive retina [22,23]. At the cellular level, the lens is surrounded by a basement membrane or capsule containing an anterior monolayer of epithelial cells that divide and terminally differentiate throughout life into highly elongated fiber cells precisely organized into tightly packed, concentric layers or growth shells to form the refractive mass (nucleus and cortex) of the lens [24,25]. Lens fiber cell differentiation is characterized by cytoplasmic accumulation of crystallin proteins, plasma membrane specialization including gap-junction plaques, actin cytoskeleton remodeling, programmed organelle loss, and core syncytium formation [24,26,27,28,29]. EPHA2 is an abundant component in the lens cell-membrane proteome accounting for ~10% of cell signaling molecules [30]. Disruption of the mouse EPHA2 gene (*Epha2*) has been associated with a variable lens phenotype ranging from severe progressive cataract formation and lens rupture to subtle nuclear opacities or clear lenses with translucent regions resulting from lens cell disorganization [20,31,32,33,34,35,36]. Here, we characterize the lens phenotype and gene expression profile of the first mice, to our knowledge, harboring mutations in the TK domain of EPHA2.

## 2. Materials and Methods

### 2.1. Mice and Lenses

*Epha2*-null mice (Stock no. 006028) [37], transgenic tandem-dimer (td)-Tomato (tdT) reporter mice (Stock no. 007576) [38], and C57BL/6J (B6J) mice (Stock no. 000664) were obtained from The Jackson Laboratory (Bar Harbor, ME, USA). Germline *Epha2*-mutant mice were generated by clustered regularly interspersed short palindromic repeats and CRISPR-associated protein 9 (CRISPR/Cas9) gene editing technology in our Genome Engineering and iPSC Center (GEiC) and Mouse Embryo Stem (ES) Cell Core facility using standard protocols as described [39]. Briefly, guide RNAs (gRNAs) were designed in silico flanking the target site and selected based on minimum off-target sites and distance from target site. A donor single-stranded oligo-deoxynucleotide (ssODN) was designed to introduce a non-synonymous (p.R722Q) variant into exon-13 of *Epha2* to replicate that (p.R721Q) predicted to be associated with age-related cortical cataract in humans [20]. Sequences of the gRNAs and ssODN were as follows:

*Epha2*.g2, 5′-gttcagtgtacttcagttggngg; Epha2.g3, 5′-ttcagtgtacttcagttggtngg; Epha2.ssODN-antisense, 5′-ggc cag gtc ccg gtg cac gta gtt cat gtt ggc cag gta ctt cat gcc gga tgc gat acc ctg cag cat gcc cac tag ctg aag tac act gaa ctc acc atc ctt ctc ctg cag aga tag gcc ctc agt gct gac cgg. Correctly edited and ‘off-target’ founder mice were identified by PCR-amplification and Sanger sequencing with gene-specific primers (Table S1, Figure S1) as described [39] and subsequently bred onto the B6J genetic background to avoid a deletion mutation in the gene for lens beaded-filament-structural-protein-2 (CP49) carried by some inbred strains [40]. *Epha2*-null mice (B6J background) were genotyped by PCR-amplification as described [34]. *Epha2*-mutant mice were crossed with tdT-reporter mice (B6J background) to generate mutant and wild type littermates that constitutively express membrane-targeted tdT. Expression of tdT was detected in vivo by means of a Dual Fluorescent Protein Flashlight (Nightsea, Lexington, MA, USA) and confirmed by PCR genotyping as described [38]. Mice were humanely killed by CO_2_ asphyxiation followed by cervical dislocation or decapitation. Eyes were removed from age- and sex-matched littermates and lenses dissected and imaged as described [35,39,41]. All mouse studies were approved by the Institutional Animal Care and Use Committee (IACUC) at Washington University (Protocol No. 20190175) in compliance with the Institute for Laboratory Animal Research (ILAR) guidelines.

### 2.2. Whole-Mount Imaging of tdT Labeled Lenses

Lenses labeled with membrane-targeted tdT were mounted in agarose-coated petri-dishes overlaid with pre-warmed cell culture medium and imaged (‘multi-area time-lapse’ function) at various depths (10–400 µm) from the lens surface using a water immersion objective lens attached to a confocal, fluorescence microscope (FluoView FV1000, Olympus, Center Valley, PA, USA) as described [35].

### 2.3. Immunofluorescence Confocal Microscopy

Enucleated eyes were processed using standard formaldehyde-fixed-paraffin-embedded (FFPE) and frozen section techniques followed by immunolabeling with antigen-specific primary antibodies (Table S2) and species-appropriate Alexa Fluor 488- or 546-conjugated secondary antibodies, (Thermo Fisher Scientific, Waltham, MA, USA), counterstaining of cell nuclei with 4′,6-diamidino-2-phenylindole (DAPI, MilliporeSigma, Burlington, MA, USA), and visualization by confocal imaging (FV1000 microscope, Olympus) performed as described [35,39,41,42].

### 2.4. Immunoblot and Immunoprecipitation Analyses

Following lens homogenization (Bullet Blender, Next Advance, Troy, NY, USA), lens post-nuclear lysates were quantified (Non-interfering protein assay, G-Bioscience, St. Louis MO, USA) and subjected to SDS-PAGE separation (Novex 4–12% gradient gels and an Invitrogen XCell electrophoresis/blot system, Thermo Fisher Scientific) and immunoblot analysis (Odyssey Infrared Imaging System, Li-Cor, Lincoln, NE, USA) using appropriate primary antibodies (Table S2) and IRDye labelled secondary antibodies (LiCor) as described [39,42]. Immunoprecipitation was performed using the Pierce Classic IP Kit (#26146, Thermo Fisher Scientific) according to the manufacturer’s instructions. Briefly, lens soluble protein (500 µg) in protease-phosphatase-inhibitor cocktail was precleared (1 h, 4 °C) with Control Agarose Resin and then serially incubated with primary antibody (10 µg, 16 h, 4 °C) followed by Protein A/G Agarose (1 h, 4 °C) to form immune complexes and the resulting eluted proteins subjected to immunoblot analysis as above with appropriate primary antibodies (Table S2).

### 2.5. RNA Sequencing Analysis

Lens total RNA was prepared in triplicate (6 lenses per sample) using the RNeasy Kit (Qiagen, Valencia, CA, USA), quantified by spectrophotometry (ND-2000, NanoDrop, Wilmington, NJ, USA) and then sized for quality by electrophoresis (2100 Bioanalyzer, Agilent Technologies, Santa Clara, CA, USA) prior to next-generation sequencing in our Genome Technology Access Center (GTAC). Samples with RNA integrity number (RIN) values >8.0 were subjected to poly-A selection (oligo-dT), chemical fragmentation, random hexamer priming, cDNA synthesis, and adapter-ligation using the TruSeq RNA Library Prep Kit (Illumina, San Diego, CA, USA) followed by paired-end (2 × 101 nt), multiplexed sequencing (HiSeq 2500, Illumina) according to the manufacturer’s instructions. Raw data were mapped to the mouse mm10 genome build using STAR (v2.5.3a) [43]. Aligned reads were filtered for quality using SAMtools (v1.4.1) [44] and read counts per gene were determined using HTSeq (v0.11.0) [45]. The EdgeR [46] was used to implement the quantile-adjusted conditional maximum likelihood method to perform pairwise comparisons and *p*-values were adjusted using the Benjamini–Hochberg Procedure. Heatmaps and other plots were generated using custom scripts and gplots, ggplot2, and ComplexHeatmap [47]. Gene ontology was performed using the Gene Ontology Resource (http://geneontology.org/; accessed on 28 July 2021) [48].

## 3. Results

### 3.1. Epha2-Mutant Mice and Lenses

**Figure 1:**
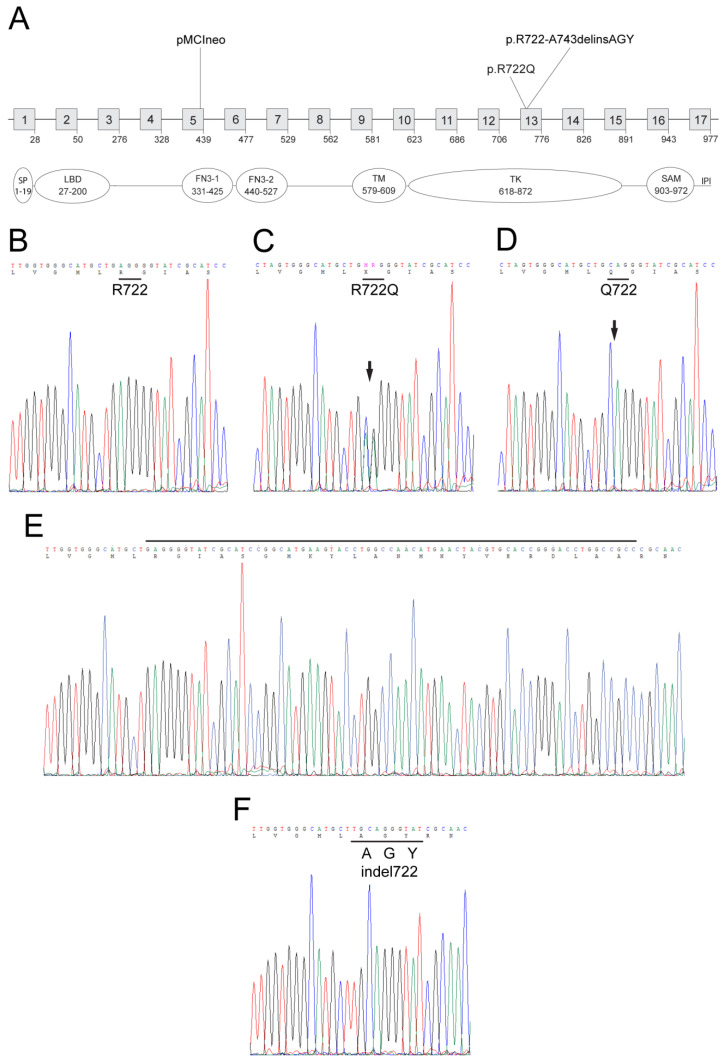
Generation of *Epha2*-mutant versus *Epha2*-null mice. (**A**) Schematic showing the exon organization and protein domains of Epha2 (RefSeq GRCm39, NM_010139.3) located on mouse chromosome 4 (physical location 141028532..141056695) spanning ~28 kb with 17 coding exons (boxes 1-17). The locations of the p.R722Q variant and the insertion-deletion mutation (exon-13) in the TK domain relative to the insertion site (exon-5) of the plasmid vector (pMCIneo) used to generate the null allele are indicated. Protein domains: SP, signal peptide, LBD, ligand-binding domain, FN3-1/2, fibronectin type-III 1 and 2, TM, transmembrane, TK—tyrosine kinase, SAM—sterile alpha-motif, IPI—PSD95/Dlg/ZO-1 (PDZ) binding motif. (**B**–**F**). Genomic DNA sequence of exon-13 showing the targeted p.R722Q missense variant in heterozygous *Epha2*-Q722 mice (**C**) and homozygous *Epha2*-Q722 mice (**D**) and the homozygous near-target insertion-deletion mutation in *Epha2*-indel722 mice (**F**) compared with the corresponding wild-type regions (**B**,**E**).

Using CRISPR/Cas9 gene editing, we generated mice to model a missense variant (c. 2162G > A, rs116506614) in exon 13 of *EPHA2* resulting in a conservative substitution of arginine-to-glutamine (p.Arg721Gln or p.R721Q) in the TK domain of EPHA2 that has been associated with age-related cortical cataract in humans [20]. Amino acid alignment revealed that the R721 codon (CGG) in human *EPHA2* was phylogenetically conserved with the R722 codon (AGG) of mouse *Epha2*. A donor single-stranded oligonucleotide was designed to introduce a two base pair change (c.2164_2165delAG > CA) that converted R722 (AGG) to Q722 (CAG). Genomic PCR and Sanger sequencing confirmed the introduction of the correct A > C transversion and G > A transition in exon-13 of *Epha2* in 9 of 47 (19%) founder (F0) mice. However, six of the nine F0 mice had acquired additional ‘off-target’ sequence changes including insertions and/or deletions (data not shown) leaving three (6%) correctly targeted mice (one male homozygote and two female heterozygotes) that were crossed to generate homozygous F1 offspring. Sequencing confirmed germline transmission of the p.R722Q substitution (Figure 1). Both heterozygous (p.R722Q) and homozygous (p.Q722Q) mutants were viable and fertile with no obvious signs of gross anatomical or behavioral abnormalities.

In addition to the correctly targeted mutant mice, we found that one of the off-target F0 mice was heterozygous for a novel *Epha2* mutation. At the DNA level this mutant allele comprised a single base insertion (c.2162_2163insT) causing a reading frame-shift that introduced three novel codons including the targeted 2-nucleotide change (GCA GGG TAT) followed by deletion of a 58-bp coding region before returning to the correct reading-frame 19 codons downstream (at codon R744). At the protein level, this compound mutation was predicted to cause an in-frame replacement of a 22-amino acid region (722-RGIASGMKYLANMNYVHRDLAA-743) from the TK domain of EPHA2 with three novel residues (722-AGY-724) resulting in an insertion-deletion mutation (p.R722_A743delinsAGY) that ‘truncates’ the wild type protein from 977 amino acids to 958 amino-acids (Figure 1). As with correctly targeted mice, sequencing confirmed germline transmission of this insertion-deletion (indel722) allele (Figure 1). Both heterozygous and homozygous F1 indel722 mutants were viable and fertile with no obvious anatomical or behavioral abnormalities.

**Figure 2:**
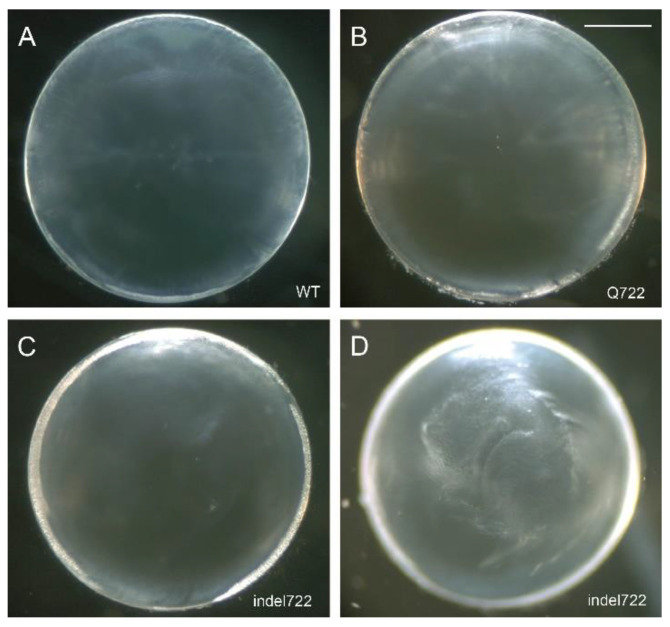
*Epha2*-mutant lens phenotype. (**A**–**D**) Representative dissecting-microscope images of wild-type (**A**), *Epha2*-Q722 (**B**) and *Epha2*-indel722 (**C**,**D**) lenses (P21) taken with the anterior pole down under dark-field illumination focused either on the lens periphery or equator region (**A**–**C**) or the posterior polar surface (**D**). Scale bar: 500 µm.

Enucleated eyes from heterozygous and homozygous *Epha2*-mutant mice (Q722 and indel722) appeared indistinguishable from wild type and dissected lenses of heterozygous *Epha2*-mutants were grossly similar to wild type at 1–2 months of age (data not shown). By contrast, homozygous indel722 mutant lenses displayed translucent regions of internal refractive disturbance that were not observed in homozygous Q722 mutant or wild type lenses (Figure 2). However, neither heterozygous nor homozygous *Epha2*-mutant lenses manifest cataract formation through 12 months of age (data not shown). All subsequent studies were performed on lenses from homozygous *Epha2*-mutants referred to as *Epha2*-Q722 and *Epha2*-indel722 throughout.

### 3.2. Lens Cell Alignment and Suture Formation in Epha2-Mutant Mice

**Figure 3:**
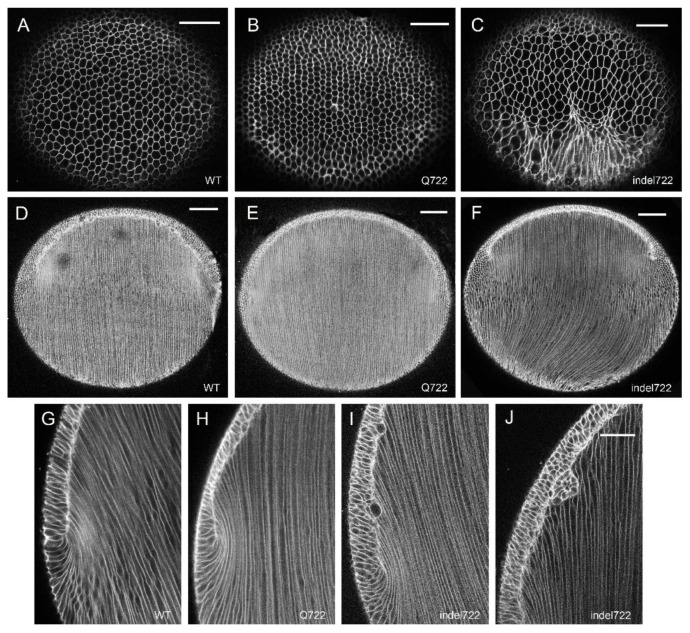
Whole-mount imaging of epithelial-to-fiber cell alignment in *Epha2*-mutant lenses. Representative superficial (10–20 µm depth) equatorial images (**A**–**C**), intermediate (100–150 µm depth) equatorial images (**D**–**F**), and deep (300–400 µm depth) equatorial images (**G**–**J**) of wild-type (**A**,**D**,**G**), *Epha2*-Q722 (**B**,**E**,**H**), and *Epha2*-indel722 (**C**,**F**,**I**,**J**) lenses (P7). Scale bar: 50 µm (**A**–**C**,**G**–**J**), 100 µm (**D**–**F**).

To visualize the global cellular organization of whole lenses ex vivo from *Epha2*-mutant mice, we generated *Epha2*-mutant and wild type littermates that constitutively express the red fluorescent protein tdT on cell membranes. First, we focused on the lens equator region where anterior epithelial cells begin terminal differentiation into highly-elongated fiber cells that form the refractive mass of the lens. In the wild type tdT lens (postnatal day 7, P7), equatorial imaging near the surface (10–20 µm depth) revealed the precise alignment of elongating, hexagonal-shaped fiber cells (in cross section) into meridional rows (Figure 3A). Such meridional alignment occurs as elongating fiber cells start migrating their apical tips across the anterior epithelium toward the anterior pole and their basal tips across the posterior capsule toward the posterior pole. At intermediate equatorial depths (100–150 µm), wild type fiber cells were aligned parallel to the anterior-posterior polar (i.e., optical) axis of the lens (Figure 3D). Imaging at greater equatorial depths (350–400 µm) in the wild type tdT lens revealed the ‘fulcrum’ (Figure 3G) where the apical tips of anterior epithelial cells pivot with the apical tips of elongating fiber cells [49]. Similar equatorial imaging of *Epha2*-Q722-tdT lenses revealed epithelial-to-fiber cell alignment including meridional rows and fulcrum formation along with pole-to-pole alignment of fiber cells resembling that found in wild type (Figure 3B,E,H). By contrast, equatorial imaging of *Epha2*-indel722-tdT lenses revealed elongating fiber cells characterized by misaligned meridional rows, deviation from the polar axis particularly at the posterior pole, and less sharply defined fulcrum formation with abnormal epithelial cell gaps and clustering (Figure 3C,F,I,J). We note that our attempts to image tdT-labelled lenses prior to P7 were hampered by their tendency to rupture during removal of the surrounding vasculature that is highly autofluorescent and interferes with imaging of these small lenses.

**Figure 4:**
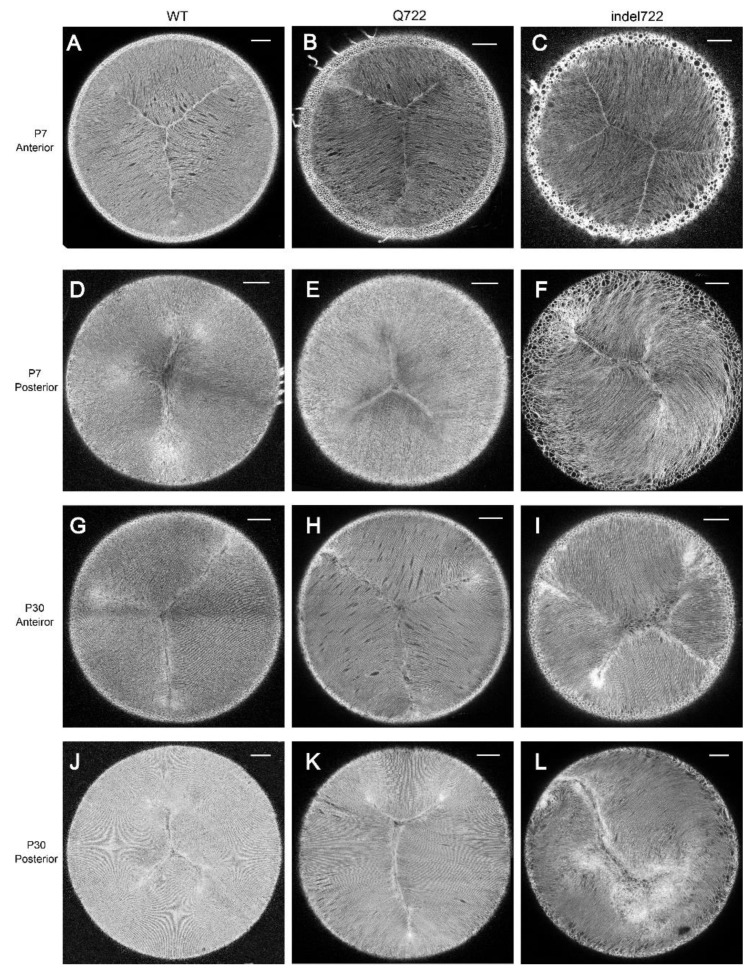
Whole-mount imaging of Y-suture formation in *Epha2*-mutant lenses. (**A**–**C**,**G**–**I**) Representative anterior suture images of wild type (**A**,**G**), *Epha2-Q722* (**B**,**H**), and *Epha2*-indel722 (**C**,**I**) lenses at P7 (**A**–**C**) and P30 (**G**–**I**). (**D**–**F**,**J**–**L**) Representative posterior suture images of wild-type (**D**,**J**), *Epha2-Q722* (**E**,**K**), and *Epha2*-indel722 (**F**,**L**) lenses at P7 (**D**–**F**) and P30 (**J**–**L**). Image depth from lens surface: 100–150 µm (**A**–**L**). Scale bar: 100 µm.

We next focused on the anterior and posterior pole regions of the lens where the tips of elongating fiber cells converge and overlap to form virtual, 3-branch (Y-shaped), suture lines centered on the optical axis [34,50,51]. In the wild type tdT lens (at P7 and P30), polar imaging revealed upright Y-shaped anterior suture lines and inverted Y-shaped posterior suture-lines that were rotationally spaced ~120° apart and centered on the optical axis (Figure 4A,D,G,J). Similarly, polar imaging of the *Epha2*-Q722-tdT lens, revealed anterior upright Y-sutures that resembled those of wild type (Figure 4B,H). However, posterior sutures at P30 were more variable than wild type with a tendency to form longer and/or unequal suture branches (Figure 4K). By contrast, polar imaging of the *Epha2*-indel722-tdT lens revealed variable disturbance of the anterior sutures often forming double-Y shapes (Figure 4C,I), whereas the posterior sutures were severely disturbed with groups of locally aligned fiber cells veering away from the optical axis to form offset, irregular, cleft-like formations with no discernable pattern or orientation (Figure 4F,L). Overall, these imaging data support a role for EPHA2 signaling in epithelial-to-fiber cell alignment (meridional row and fulcrum formation) at the lens equator and suture formation at the lens poles.

### 3.3. Expression and Distribution of EPHA2 Mutants in the Lens

**Figure 5:**
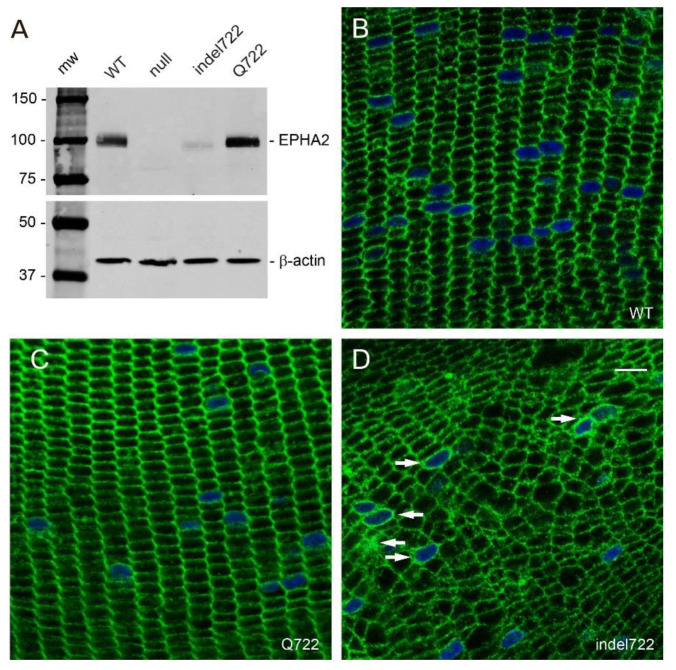
Expression and distribution of EPHA2 mutants in the lens. (**A**) Immunoblot analysis of *Epha2*-mutant lenses. (**B**–**D**). Immuno-localization of EPHA2 in wild type (**B**), *Epha2*-Q722 (**C**), and *Epha2*-indel722 (**D**) mutant lenses (P21). Arrows in panel D indicate intracellular and/or perinuclear localization of EPHA2. Scale bar, 10 µm.

To determine the effects of the Q722 and indel722 mutations on the expression and distribution of EPHA2 and other lens cell membrane proteins, we performed immunoblot analysis and immunofluorescence confocal microscopy. Immunoblotting revealed that the Q722 mutant was expressed at levels similar to wild type EPHA2 in the lens, whereas the indel722 mutant was present at reduced levels compared to the Q722 mutant and migrated with a molecular mass slightly lower (~2 kDa) than wild type EPHA2 (Figure 5A). These data are consistent with the in-frame deletion of 19 amino acids from the TK domain of EPHA2 (Figure 1) and suggest that the ‘truncated’ indel722 mutant protein and/or transcript may be relatively unstable compared to the full-length Q722 mutant in the lens. However, we cannot exclude reduced affinity and/or avidity of the EPHA2 antibody for the indel722 mutant versus the Q722 mutant on immunoblots.

In the wild type lens, immunofluorescent labeling revealed that EPHA2 was localized to fiber cell membranes highlighting the characteristic radial columns of flattened hexagonal cells of similar cross-sectional area serially aligned throughout the cortical region of the lens [50]—particularly along the short membrane faces (Figure 5B). Similarly, in the *Epha2*-Q722 lens, immunolabeling revealed that EPHA2 was primarily localized to radial columns of hexagonal fiber cell membranes (Figure 5C). By contrast, in the *Epha2*-indel722 lens, anti-EPHA2 labeling revealed that the radial columns of hexagonal fiber cells were profoundly disorganized, particularly in the inner cortex (Figure 5D). Instead of flattened hexagons, most *Epha2*-indel722 lens fiber cells exhibited an irregular cross-sectional size and shape including 4- or 5-sided cells that were randomly arranged throughout the cortex. Further, while EPHA2 was localized to fiber cell membranes there was reproducible evidence of diffuse membrane and perinuclear labeling suggesting that at least some of the indel722 mutant was retained in one or more cytoplasmic compartments—consistent with impaired targeting to the cell surface.

**Figure 6:**
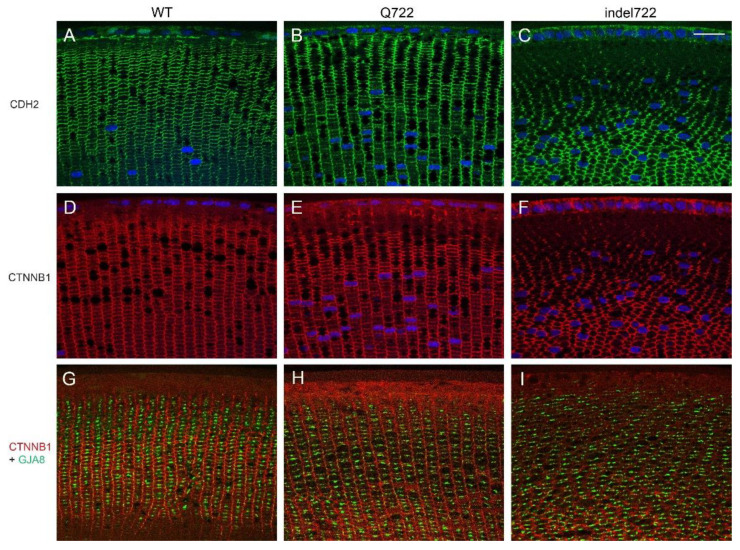
Distribution of CDH2, CTNNB1, and GJA3 in *Epha2*-mutant lenses. Immuno-localization of CDH2 (**A**–**C**), CTNNB1 (**D**–**I**), and GJA3 (**G**–**I**) in wild type (**A**,**D**,**G**), *Epha2*-Q722 mutant (**B**,**E**,**H**) and *Epha2*-indel722 mutant (**C**,**F**,**I**) lenses (P28). Scale bar, 20 µm.

To visualize the effects of *Epha2*-mutants on the distribution of other lens cell membrane proteins we performed immunofluorescent labeling with antibodies to N-cadherin or cadherin-2 (CDH2), β-catenin or catenin β1 (CTNNB1), and connexin-46 or gap-junction alpha-3 protein (GJA3). In wild type lenses, both CDH2 and CTNNB1 labeling outlined the radial columns of flattened hexagonal fiber cells particularly along the narrow membrane faces (vertical plane), whereas GJA3 was localized to gap-junction plaques particularly on the broad faces of fiber cell membranes (horizontal plane) generating a ‘ladder-like’ appearance along the radial cell columns (Figure 6A,D,G). Immunolabeling of *Epha2*-Q722 lenses confirmed that CDH2, CTNNB1, and GJA3 were localized to fiber cell membranes aligned in radial columns similar to wild type (Figure 6B,E,H). In *Epha2*-indel722 lenses, all three proteins were primarily localized to fiber cell membranes that were grossly disorganized lacking radial column alignment and, in places, deviating away from the optical plane of the lens section (Figure 6C,F,I). Overall, these data suggest that while fiber cell membrane integrity was maintained in *Epha2*-mutant lenses, the radial column patterning of flattened, hexagonal, fiber cells was profoundly disturbed in *Epha2*-indel722 lenses.

### 3.4. EPHA2 Complex Formation and Phosphorylation in the Lens

**Figure 7:**
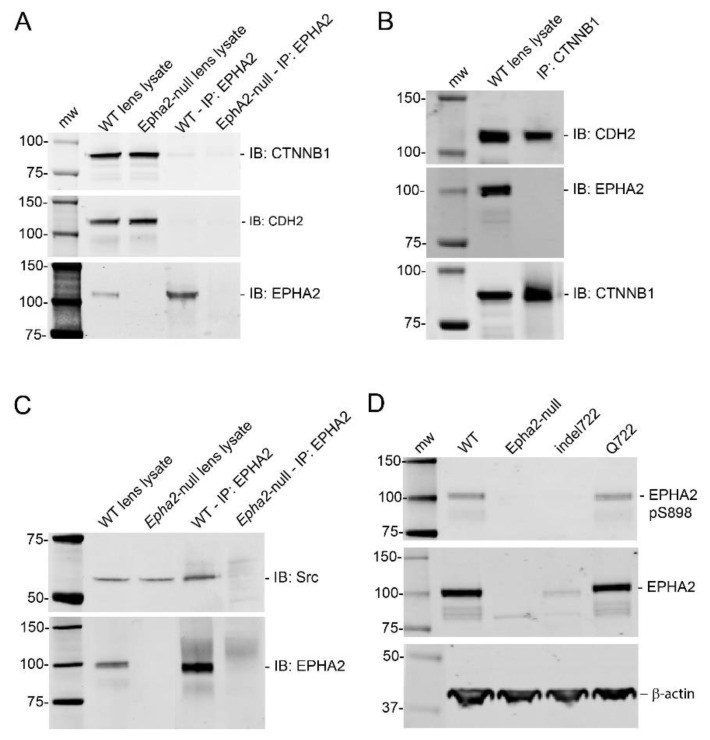
EPHA2 complex formation and phosphorylation status in the lens. (**A**–**C**) Immunoprecipitation analysis of wild type lenses (P21) showing that EPHA2 forms trace complexes with CTNNB1 but not with CDH2 (**A**) and that CTNNB1 complexes strongly with CDH2 but not with EPHA2 (**B**), whereas EPHA2 forms complexes with Src (**C**). (**D**) Immunoblot showing EPHA2 serine 898 phosphorylation (pS898) levels detected in wild-type and *Epha2*-mutant lenses.

As EPHA2 has been reported to form complexes with CTNNB1 and CDH2 [52,53] and to elicit downstream signaling in the lens through the neuronal proto-oncogene tyrosine-protein kinase Src [32], we undertook immunoprecipitation and immunoblotting techniques to detect lens EPHA2 interactions and phosphorylation status. Immunoprecipitation revealed that EPHA2 failed to form strong complexes with CDH2 and exhibited only trace complex formation with CTNNB1 relative to that between CTNNB1 and CDH2 (Figure 7A,B). By contrast, EPHA2 formed strong complexes with Src kinase (Figure 7C). Since the Q722 and indel722 mutations were located within the tyrosine-kinase domain of EPHA2 (amino acid residues 618–872), we attempted to compare the phosphorylation status of EPHA2 in wild type versus *Epha2*-mutant lenses. Phosphorylation of human EPHA2 has been reported at several tyrosine residues including Y588 (Y589 in mouse) and at several serine residues including S897 (S898 in mouse) in certain cancer cell lines [4,10,54]. EPHA2-S897/898 phosphorylation was readily detected by immunoblotting in both wild type and *Epha2*-Q722 lenses but not in *Epha2*-indel722 lenses—likely due to reduced levels of mutant protein in the latter (Figure 7D). By contrast, immunoblotting failed to detect EPHA2-Y588/589 phosphorylation in wild-type and *Epha2*-mutant lenses (data not shown). However, we are cautious about interpreting such negative results as immunoblotting of whole lenses may lack the sensitivity required to detect tyrosine phosphorylation that may be restricted to a specific sub-region of the lens (e.g., anterior epithelium). Overall, these data suggest that EPHA2 forms complexes with Src and is abundantly serine phosphorylated in the lens.

### 3.5. Gene Expression Profiles of Epha2-Mutant and Epha2-Null Lenses

**Figure 8:**
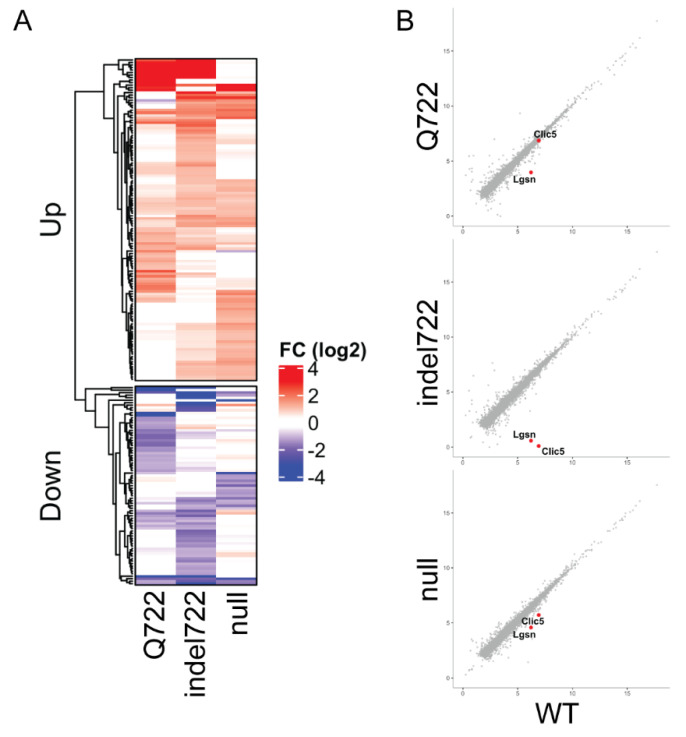
Gene expression changes in *Epha2*-mutant and *Epha2*-null lenses (P7). RNA-seq analysis identifies unique expression changes in *Epha2*-mutant (Q722, indel722) and *Epha2*-null lenses compared to wild type (**A**). Genes known to be involved in lens cell differentiation, *Lgsn* and *Clic5*, show varied downregulation across *Epha2* genotypes (**B**).

To determine the effects of the *Epha2*-Q722 and *Epha2*-indel722 mutations on lens gene expression, we performed RNA-seq analysis to compare global transcriptional changes in *Epha2*-mutant versus *Epha2*-null and wild type lenses at an early stage of phenotype development (P7). RNA-seq data files have been deposited in the Gene Expression Omnibus (GEO) database under accession no. GSE181358. Biological triplicate samples were sequenced to a depth of >20 M reads and aligned to the mouse mm10 genome build with >98% alignment rate. Differential expression analysis using EdgeR (fold-change, FC ≥ 2, false discovery rate, FDR ≤ 0.05) was performed for each *Epha2* genotype compared to wild-type (Figure 8). RNA-seq data showed high consistency between biological triplicates, with data from each *Epha2* genotype clustering independent from other samples (Figure S2A). We next analyzed the average fold-change (mutant/null vs. wild-type) within each *Epha2* genotype of all significantly affected genes (FC ≥ 2, FDR ≤ 0.05) and found that they displayed a largely unique set of dysregulated genes compared to wild type (Figure S2B). Overall, the total number of differentially expressed genes for each genotype was 86 in *Epha2*-Q722 lenses (48 up and 38 down, Table S3), 110 in *Epha2*-indel722 lenses (68 up and 42 down, Table S4), and 89 in *Epha2*-null lenses (68 up and 21 down, Table S5). We note that *Epha2* was among the downregulated genes in *Epha2*-indel722 lenses (Figure S2B, Table S4) suggesting that the in-frame insertion-deletion mutation (in exon-13) may decrease transcript stability. However, there were few other genes that displayed discordant expression between *Epha2* genotypes (Figure 8A and Figure S2B) and many genes up- or downregulated in one *Epha2* genotype were generally not similarly affected in the other genotypes. Gene ontology (GO) enrichment analyses of each list of up or down regulated genes within an individual *Epha2* genotype failed to yield any enriched categories or common pathways (data not shown). GO analysis of the combined upregulated gene set (Figure 8A and Figure S2B) showed some enrichment for genes involved in extracellular matrix structure, chloride and gated channel activity, and calcium ion binding (Figure S3), whereas the combined downregulated genes did not reveal any common pathways (data not shown). However, we note the shared downregulation of *Lgsn* and *Clic5* (Figure 8B, Tables S3–S5) both of which have been implicated in lens cytoskeletal differentiation [55,56].

## 4. Discussion

In this study, we have demonstrated that mice homozygous for mutations (Q722 or indel722) in the tyrosine kinase domain of EPHA2 underwent variable changes in lens cell organization and gene expression. *Epha2*-Q722 mice displayed clear lenses with mild defects in Y-suture branching at the posterior pole, whereas *Epha2*-indel722 mice presented clear lenses with translucent regions resulting from severe disturbance of (1) epithelial-to-fiber cell alignment (meridional row and fulcrum formation) at the lens equator, (2) radial cell column formation throughout the lens cortex, and (3) Y-suture branching at the lens poles—similar to those described for *Epha2*-null lenses [35]. As meridional row and fulcrum formation were already disturbed at P7, it is conceivable that cell patterning defects may have arisen during earlier stages of lens development. EPHA2 was mainly localized to radial columns of hexagonal fiber cell membranes throughout the cortex of *Epha2*-Q722 lenses, whereas fiber cell columns were severely disorganized in *Epha2*-indel722 lenses along with cytoplasmic retention of EPHA2—consistent with failed targeting to the cell surface. EPHA2 formed strong immuno-complexes with Src kinase in vitro supporting a role for EPHA2/Src signaling during lens development [32]. However, we were unable to replicate strong EPHA2 complexes with CTNNB1 or CDH2 in the lens at wean-age (P21) similar to those reported in transfected (293T) cells and in the lens at an earlier stage of postnatal development (P10) [52,53]. EPHA2 was abundantly phosphorylated on serine-897/898 in wild type and *Epha2*-Q722 mutant lenses (P21), whereas EPHA2 tyrosine588/589 phosphorylation was not detected using similar immunoblot analysis of whole lenses. The relative abundance of serine-897/898 phosphorylation in the lens suggests that ephrin-independent or non-canonical EPHA2 signaling [57] may participate in lens cell migration. However, we cannot exclude a role for ephrin-dependent or canonical EPHA2 signaling since the hallmark tyrosine-588/589 phosphorylation may be restricted to specific sub-regions of the lens (e.g., specific lens epithelial cells) requiring more detailed studies. At the transcript level, several genes encoding cytoskeletal-associated proteins were differentially regulated including shared downregulation of *Lgsn* in both *Epha2*-mutant and *Epha2*-null lenses and *Clic5* in *Epha2*-indel722 and *Epha2*-null lenses. Combined, our imaging and transcript data support a role for EPHA2 signaling—potentially via the cytoskeleton—in generating the precise cellular patterning underlying the refractive properties and optical quality of the crystalline lens.

Functional (over)expression studies in cultured (transfected) cell-lines have been used to predict diverse pathogenic mechanisms underlying EPHA2-related forms of human cataract. A non-coding risk allele for age-related cataract (rs6603883) located in a paired-box-2 (PAX2) binding-site within the EPHA2 gene promoter suggested that it acts by down-regulating EPHA2 expression in cultured lens cells [58]. Several SAM domain mutations underlying early-onset cataract were reported to alter receptor stability, function and/or sub-cellular distribution [59,60,61]. Of three missense variants located within the TK domain of EPHA2 (amino acid residues 613–871), two (p.G668D, p.Q669H) have been associated with early-onset cataract and one (p.R721Q) with age-related cortical cataract in humans [20,62,63]. The p.G668D mutant has been associated with increased proteasome-mediated degradation, altered subcellular localization, and increased cell migration [63], whereas the p.R721Q mutant was associated with increased basal kinase activation in the absence of ligand, inhibition of clonal cell growth, and variable intracellular retention [20]. In our mouse model of the human EPHA2-p.R721Q variant (*Epha2*-Q722), homozygous expression of the equivalent variant protein at constitutive levels resulted in mild disturbance of the posterior Y-sutures but not in early-onset or age-related cataract (Figure 2 and Figure 4). Similarly, homozygous expression of an in-frame TK domain mutant did not elicit cataract development in *Epha2*-indel722 lenses despite decreased levels and cytoplasmic retention of the mutant protein coupled with severe disorganization of lens fiber cells causing translucent regions of poor optical quality (Figure 2). While there was some mechanistic agreement between in vitro (overexpression) and in vivo (constitutive) expression studies of EPHA2 mutants (e.g., intracellular retention and altered cell growth/migration), we cannot account specifically for the lack of cataract penetrance in the *Epha2*-mutant mice reported here. Contributing factors include species differences in genetic background modifier effects, variable environmental risk factors (e.g., UV exposure in nocturnal mice versus diurnal humans), and morphological differences between the relatively small, almost spherical mouse lens with Y-suture branching versus the much larger, ellipsoidal human lens with more complex star-suture branching [51].

While we did not observe cataract formation in *Epha2*-mutant (Q722, indel722) or *Epha2*-null lenses [35], there were significant changes in lens gene expression at the transcript level between *Epha2* genotypes as early as P7. Among the most upregulated genes (>4-fold) in both *Epha2*-Q722 and *Epha2*-indel722 mutant lenses were those for tubulin alpha 1C (TUBA1C) and alkaline ceramidase-2 (ACER2). TUBA1C serves as a prognostic biomarker for a variety of cancers [64] and ACER2 is a Golgi enzyme involved in regulating B1 integrin maturation and cell adhesion [65]. In *Epha2*-Q722 and *Epha2*-null lenses, the gene for steroidogenic acute regulatory protein-related lipid transfer (START) domain-containing protein 9 (STARD9) was strongly upregulated, whereas that for doublecortin domain-containing 2a (DCDC2a) was strongly upregulated in *Epha2*-indel722 and *Epha2*-null lenses. STARD9 functions as a centrosomal protein that regulates both interphase and mitotic spindle microtubules [66], whereas DCDC2a serves as a micro-tubule associated protein localized to hair cell kinocilia and supporting cell primary cilia that when mutated causes non-syndromic recessive deafness in humans [67]. The most consistently upregulated gene in both *Epha2*-mutant and *Epha2*-null lenses was that for WD-repeat and FYVE-domain-containing protein-1 (WDFY1), which serves as an adapter protein in toll-like receptor signaling [68]. Finally, the gene for dorsal inhibitory axon guidance protein (DRAXIN) was strongly upregulated in *Epha2*-indel722 lenses and that for actin, alpha 2, smooth muscle, aorta (ACTA2) was moderately upregulated in *Epha2*-null lenses. While ACTA2 serves as a marker for epithelial–mesenchymal transition during cataract formation [69] and several of the other upregulated genes share cytoskeletal-related or signaling functions, none have yet been associated with EPHA2 signaling or lens cell differentiation.

Among the most downregulated genes, two have been directly implicated in lens-specific cytoskeleton biology. The most consistently downregulated gene in *Epha2*-Q722 (>−4-fold), *Epha2*-indel722 (>−100-fold), and *Epha2*-null (>−3-fold) lenses was that for lens glutamine synthase-like or lengsin (LGSN), also known as glutamate-ammonia ligase (glutamine synthase) domain containing 1 (GLULD1), a lens-specific protein with a glutamine synthase domain lacking glutamine synthase activity [55]. LGSN is a late marker for lens fiber cell terminal differentiation and has been shown to co-localize with actin and interact with the lens-specific intermediate filament protein, beaded filament structural protein-2 (BFSP2), also known as cytoskeletal protein 49 (CP49) or phakinin, suggesting that LGSN represents a recruited enzyme adapted to act as a cytoskeletal component or chaperone during remodeling of the lens cytoskeleton [55,70].

The most downregulated gene in *Epha2*-indel722 mutant lenses (<−1000-fold), and to a lesser extent in *Epha2*-null lenses (<−2-fold), was that for chloride intracellular channel 5 (CLIC5). Mutations in the human CLIC5 gene have been linked with progressive autosomal recessive, non-syndromic sensorineural hearing impairment with or without vestibular dysfunction and CLIC5 was found to be abundantly expressed in the fetal inner ear [71,72]. Similarly, in *jitterbug* (*jbg*) mice a spontaneous deletion mutation in *Clic5* underlies hearing loss with vestibular and renal dysfunction and CLIC5 was localized to the base of hair cell stereocilia where it complexes with radixin, taperin, and myosin VI to stabilize cell membrane–actin cytoskeleton attachments [73]. Recently, CLIC5 been localized to cilia and/or centrosomes in the lens and *Clic5*-mutant (*jtb*) lenses were found to exhibit defective suture formation [56]. Further, EPHA2 has been shown to regulate Src/cortactin/F-actin complexes during epithelial-to-fiber cell morphogenesis (meridional row and fulcrum formation) at the lens equator [32]. Collectively, these observations point to a functional synergy between EPHA2 and several cytoskeletal proteins with LGSN and CLIC5 providing promising candidates for future studies of EPHA2 signaling in the lens.

In conclusion, our data suggest that EPHA2 signaling is required for lens cell pattern recognition and support a role for EPHA2 in cytoskeleton dynamics during lens cell differentiation.

## Data Availability

RNA-seq data files have been deposited in the Gene Expression Omnibus (GEO) database under accession no. GSE181358.

## Supplementary Materials

The following are available online at https://www.mdpi.com/article/10.3390/cells10102606/s1. Figure S1. Allele-specific PCR-genotyping of *Epha2*-mutant mice. (A) PCR amplicons of wild type (R722), heterozygous *Epha2*-Q722 (R722Q) and homozygous *Epha2*-Q722 (Q722) alleles using three exon-13 primers (Table S1) indicated by arrows in the schematic below. (B) PCR amplicons of wild type (*+/+*), heterozygous *Epha2*-indel722 (+/indel722), and homozygous *Epha2*-indel722 (indel722) alleles using exon-13 flanking primers. Figure S2. RNA-seq data differential expression analysis. Triplicate samples from wild-type (WT), *Epha2*-mutant (Q722, indel722), and *Epha2*-null lenses (P7) mostly cluster independently for all dysregulated genes (A). Full heat-map of Figure 8A displays FC of each gene relative to WT in each *Epha2* genotype tested (B). Figure S3. Gene ontogeny (GO) analysis of the combined upregulated genes from *Epha2*-mutant (Q722, indel722) and *Epha2*-null lenses (P7). Table S1. Primer sequences used for PCR-amplification and Sanger sequencing of *Epha2*. Table S2. Primary antibodies used for confocal microscopy, immunoprecipitation, and immunoblotting. Table S3. Differentially regulated genes (fold-change FC ≥ 2, false discovery rate FDR ≤ 0.05) in the *Epha2*-Q722 lens (P7). Table S4. Differentially regulated genes (fold-change FC ≥ 2, false discovery rate FDR ≤ 0.05) in the *Epha2*-indel722 lens (P7). Table S5. Differentially regulated genes (fold-change FC ≥ 2, false discovery rate FDR ≤ 0.05) in the *Epha2*-null lens (P7).
