# Supplementary material for: Mutation of the EPHA2 Tyrosine-Kinase Domain Dysregulates Cell Pattern Formation and Cytoskeletal Gene Expression in the Lens
Source: Cells. 2021 Sep 30;10(10):2606. doi: 10.3390/cells10102606 (PMC8534143; doi:10.3390/cells10102606)
Supplement: Supplementary file 1 [file cells-10-02606-s001.zip › cells-1356926-supplementary.pdf]

Mutation of the EPHA2 tyrosine-kinase domain dysregulates cell pattern formation and cytoskeletal gene expression in the lens

Yuefang Zhou, Thomas M. Bennett, Philip A. Ruzycki, and Alan Shiels*

__Supplementary Materials:__

Figure S1. Allele-specific PCR-genotyping of *Epha2*-mutant mice. (**A**) PCR amplicons of wild-type (R722), heterozygous *Epha2*-Q722 (R722Q) and homozygous *Epha2*-Q722 (Q722) alleles using three exon-13 primers (**Table 1**) indicated by arrows in the schematic below. (**B**) PCR amplicons of wild-type (+/+), heterozygous *Epha2*-indel722 (+/indel722), and homozygous *Epha2*-indel722 (indel722) alleles using exon-13 flanking primers.

Figure S2. RNA-seq data differential expression analysis. Triplicate samples from wild-type (WT), *Epha2*-mutant (Q722, indel722), and *Epha2*-null (KO) lenses mostly cluster independently for all dysregulated genes (**A**). Full heat-map of Figure 8A displays FC of each gene relative to WT in each *Epha2* genotype tested (**B**).

Figure S3. Gene ontogeny (GO) analysis of the combined upregulated genes from *Epha2*-mutant (Q722, indel722) and *Epha2*-null lenses (P7).

Table S1. Primer sequences used for PCR-amplification and Sanger sequencing of *Epha2*.

Table S2. Primary antibodies used for confocal microscopy, immunoprecipitation, and immunoblotting.

Table S3. Differentially regulated genes (fold-change FC ≥ 2, false discovery rate FDR ≤ 0.05) in the *Epha2*-Q722 lens (P7).

Table S4. Differentially regulated genes (fold-change FC ≥ 2, false discovery rate FDR ≤ 0.05) in the *Epha2*-indel722 lens (P7).

Table S5. Differentially regulated genes (fold-change FC ≥ 2, false discovery rate FDR ≤ 0.05) in the *Epha2*-null lens (P7).

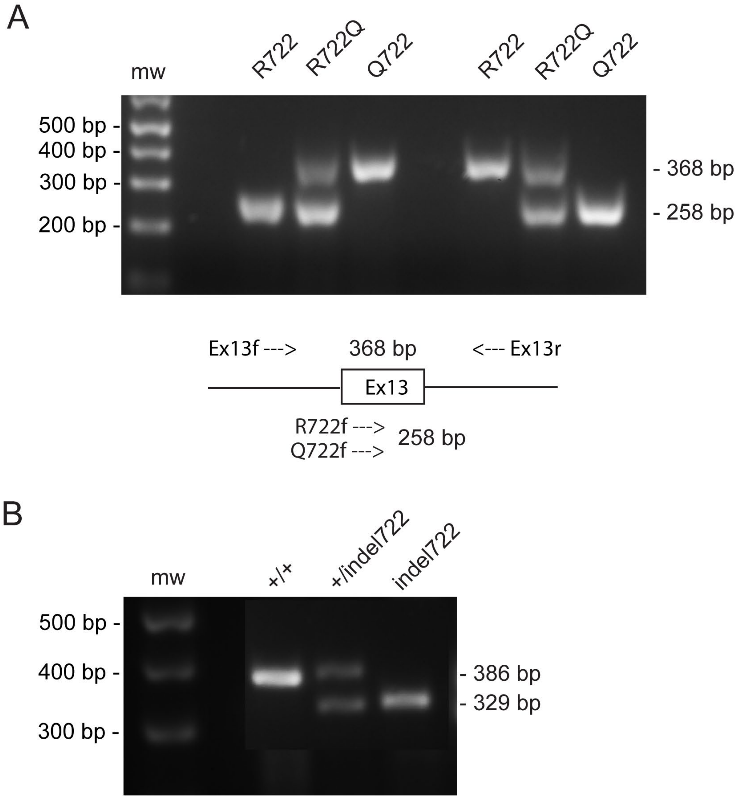

Figure S1. Allele-specific PCR-genotyping of *Epha2*-mutant mice. (A) PCR amplicons of wild-type (R722), heterozygous *Epha2*-Q722 (R722Q) and homozygous *Epha2*-Q722 (Q722) alleles using three exon-13 primers (Table 1) indicated by arrows in the schematic below. (B) PCR amplicons of wild-type (+/+), heterozygous *Epha2*-indel722 (+/indel722), and homozygous *Epha2*-indel722 (indel722) alleles using exon-13 flanking primers.

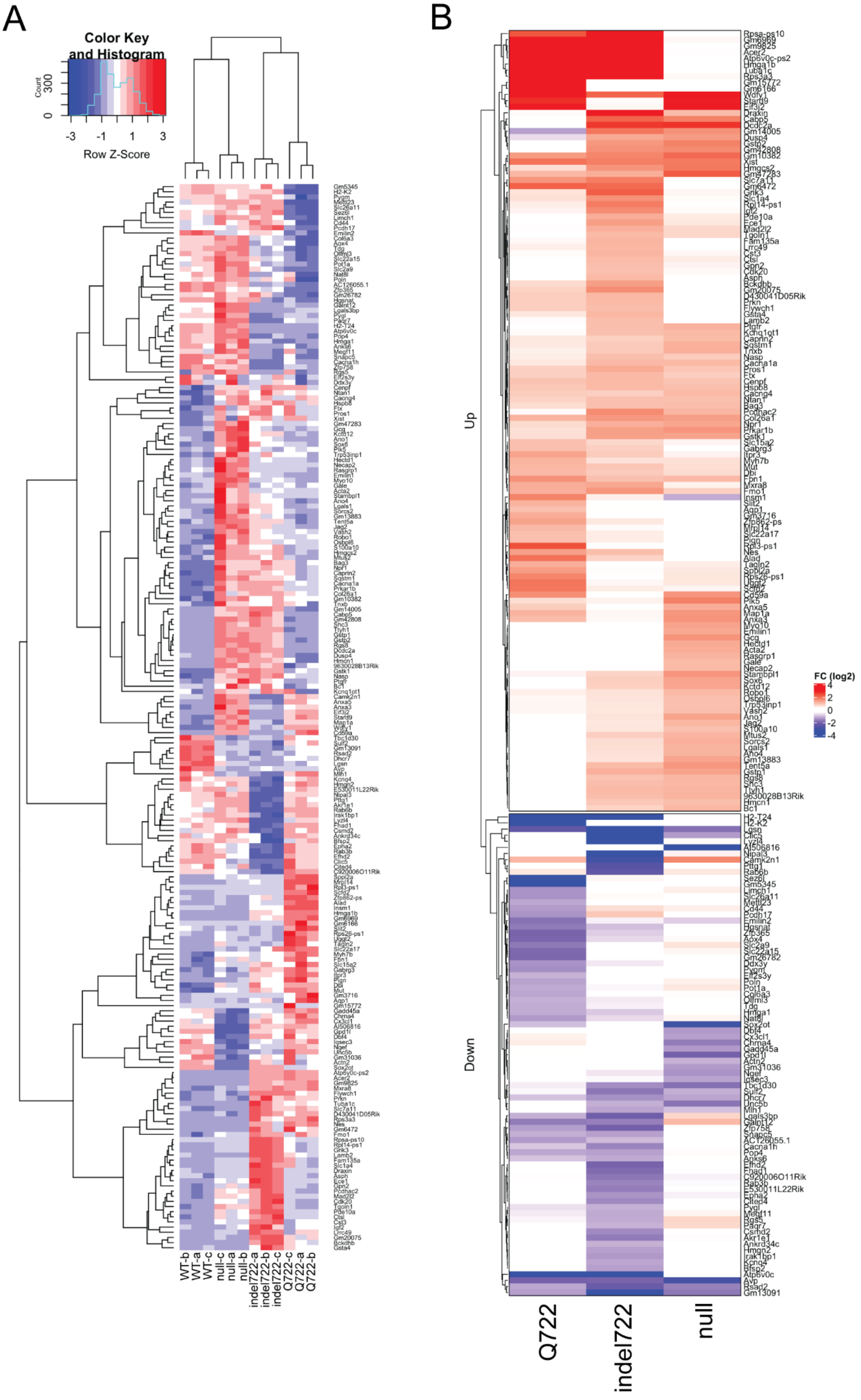

A
Color Key and Histogram
Count
0
300
-3
-1
1
3
Row Z-Score
WT-b
WT-a
WT-c
null-c
null-a
null-b
indel722-a
indel722-b
indel722-c
Q722-c
Q722-a
Q722-b
B
Up
Down
FC (log2)
4
2
0
-2
-4
Q722
indel722
null

Figure S2. RNA-seq data differential expression analysis. Triplicate samples from wild-type (WT), *Epha2*-mutant (Q722, indel722), and *Epha2*-null (KO) lenses mostly cluster independently for all dysregulated genes (**A**). Full heat-map of Figure 8A displays FC of each gene relative to WT in each *Epha2* genotype tested (**B**).

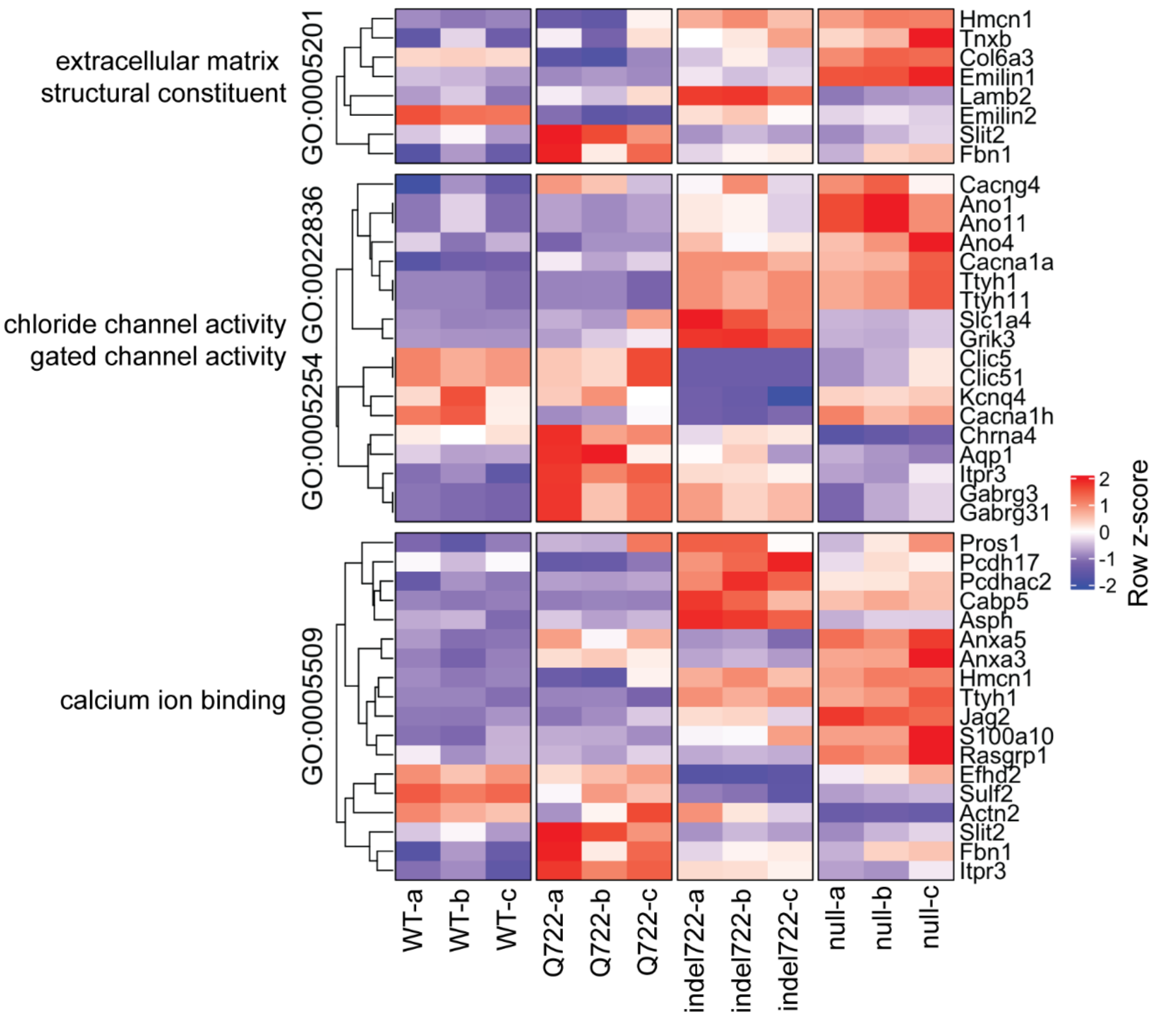

Figure S3. Gene ontogeny (GO) analysis of the combined upregulated genes from *Epha2*-mutant (Q722, indel722) and *Epha2*-null lenses (P7).

Table S1: Primer sequences used for PCR-amplification and Sanger sequencing of *Epha2*

Primer name	Location	Strand	Sequence (5' - 3')	Target	Amplicon
Mm_Epha2_Ex13F	Intron 12	Sense	tgtaaaacgacggccagtAGGGTAAGGTCATGCTCTGC	Wild-type & indel722 allele	386 bp & 329 bp
Mm_Epha2_Ex13R	Intron 13	Antisense	caggaaacagctatgaccCTGCGTCACCTCTGAGCC		
Mm Epha2 R722F	Exon 13	Sense	TCAGTTGGTGGGCATGCTGAG	R722 allele	258 bp
Mm Epha2 Q722F	Exon 13	Sense	TCAGCTAGTGGGCATGCTGCA	Q722 allele	258 bp

Universal M13 ‘tail’ sequencing primer (18-mer) shown in lowercase font.

Table S2. Primary antibodies used for immunofluorescence confocal microscopy, immunoprecipitation, and immunoblotting.

Primary antibody	Host	Clonality	Catalog #	Manufacturer
EphA2	goat	polyclonal	AF639	R&D Systems, Minneapolis, MN
EphA2 (C-20)	rabbit	polyclonal	sc-924	Santa Cruz Biotechnology, Dallas, TX
Phospho-EphA2 (Ser897)	rabbit	monoclonal	6347S	Cell Signaling Technology (CST), Danvers, MA
Phospho-EphA2 (Tyr588)	rabbit	monoclonal	12677S	Cell Signaling Technology (CST), Danvers, MA
Cadherin, N- (neural)	rat	monoclonal	MNCD2	Developmental Studies Hybridoma Bank (DSHB), Iowa City, IA
Catenin, beta	mouse	monoclonal	BD 610153	BD Biosciences, Franklin Lakes, NJ
Connexin 46 (M-19)	goat	polyclonal	sc-20861	Santa Cruz Biotechnology, Dallas, TX
Src (GD11)	mouse	monoclonal	05-184	EMD Millipore, Billerica, MA
Phospho-Src (Tyr416)	rabbit	monoclonal	6943	Cell Signaling Technology, CST, Danvers, MA
Phospho-Src (Tyr416)	rabbit	polyclonal	2101	Cell Signaling Technology, CST, Danvers, MA
β-Actin	mouse	monoclonal	3700	Cell Signaling Technology, CST, Danvers, MA

Table S3. Differentially regulated genes (FC > 2, FDR = 0.05) in the *Epha2* -Q722 lens (P7)

Gene_id	logFC.Q722.wt	FC.Q722.wt	FDR.Q722.wt	Description
**Upregulated**				
Rps3a3	6.4868	89.6857	5.69E-244	ribosomal protein S3A3
Hmga1b	5.8204	56.5072	1.16E-112	high mobility group AT-hook 1B
Tuba1c	5.4754	44.4908	1.39E-222	tubulin, alpha 1C
Gm6969	5.1602	35.7593	6.99E-136	predicted pseudogene 6969
Atp6v0c-ps2	4.6560	25.2114	2.71E-128	ATPase, H+ transporting, lysosomal V0 subunit C, pseudogene 2
Acer2	4.3259	20.0558	5.95E-245	alkaline ceramidase 2
Gm9825	4.3107	19.8446	4.44E-107	predicted gene 9825
Gm6166	4.1759	18.0741	9.15E-292	predicted gene 6166
Eif3j2	3.4050	10.5929	4.66E-53	eukaryotic translation initiation factor 3, subunit J2
Wdfy1	3.3559	10.2383	7.91E-59	WD repeat and FYVE domain containing 1
Gm15772	3.3492	10.1909	1.16E-132	predicted gene 15772
Stard9	2.6932	6.4676	1.11E-25	START domain containing 9
Rpl3-ps1	2.3346	5.0440	1.16E-59	ribosomal protein L3, pseudogene 1
Rpsa-ps10	2.2735	4.8348	9.26E-63	ribosomal protein SA, pseudogene 10
Gm6472	2.1384	4.4027	8.25E-40	predicted pseudogene 6472
Xist	1.9394	3.8355	1.33E-54	inactive X specific transcripts
Alad	1.9340	3.8210	2.59E-57	aminolevulinate, delta-, dehydratase
Uggt2	1.8883	3.7020	1.05E-24	UDP-glucose glycoprotein glucosyltransferase 2
Scfd2	1.8504	3.6061	2.02E-34	Sec1 family domain containing 2
Insm1	1.7253	3.3066	2.81E-21	insulinoma-associated 1
Rps26-ps1	1.6934	3.2341	1.09E-31	ribosomal protein S26, pseudogene 1
Cd59a	1.5833	2.9965	4.35E-27	CD59a antigen
Fbn1	1.5482	2.9246	7.58E-22	fibrillin 1
Slc7a11	1.4991	2.8267	2.63E-40	solute carrier family 7 (cationic amino acid transporter, y+ system), member 11
Gm10382	1.4377	2.7090	1.07E-09	predicted gene 10382
Sppl2a	1.4209	2.6776	4.65E-39	signal peptide peptidase like 2A
Nes	1.3879	2.6170	3.63E-22	nestin
Zfp862-ps	1.3655	2.5766	2.70E-15	zinc finger protein 862, pseudogene
Itpr3	1.3530	2.5545	9.40E-15	inositol 1,4,5-triphosphate receptor 3
Mxra8	1.2365	2.3562	3.10E-19	matrix-remodelling associated 8
Gm3716	1.2234	2.3349	3.40E-14	predicted gene 3716
Tagln2	1.2129	2.3180	1.86E-11	transgelin 2
Fmo1	1.1963	2.2915	1.04E-07	flavin containing monooxygenase 1
Slit2	1.1617	2.2372	3.96E-21	slit homolog 2 (Drosophila)
Col26a1	1.1551	2.2270	1.21E-05	collagen, type XXVI, alpha 1
Myh7b	1.1404	2.2045	5.19E-10	myosin, heavy chain 7B, cardiac muscle, beta
Map1a	1.1396	2.2032	3.21E-18	microtubule-associated protein 1 A
Mut	1.1225	2.1773	1.54E-12	methylmalonyl-Coenzyme A mutase
Gm47283	1.0999	2.1434	6.14E-08	predicted gene 47283
Camk2n1	1.0849	2.1212	6.98E-08	calcium/calmodulin-dependent protein kinase II inhibitor 1
Mrpl14	1.0846	2.1208	1.28E-12	mitochondrial ribosomal protein L14
Dbi	1.0687	2.0976	1.20E-22	diazepam binding inhibitor
Anxa3	1.0498	2.0703	3.43E-14	annexin A3
Slc22a17	1.0264	2.0370	8.14E-20	solute carrier family 22 (organic cation transporter), member 17
Slc15a2	1.0183	2.0255	3.38E-15	solute carrier family 15 (H+/peptide transporter), member 2
Gabrg3	1.0178	2.0249	6.76E-18	gamma-aminobutyric acid (GABA) A receptor, subunit gamma 3
Aqp1	1.0166	2.0232	9.68E-15	aquaporin 1
Pign	1.0152	2.0211	9.11E-12	phosphatidylinositol glycan anchor biosynthesis, class N
**Downregulated**				
Eif2s3y	-1.0081	-2.0112	1.50E-11	eukaryotic translation initiation factor 2, subunit 3, structural gene Y-linked
Olfml3	-1.0256	-2.0357	2.67E-19	olfactomedin-like 3
Lgals3bp	-1.0438	-2.0617	1.33E-07	lectin, galactoside-binding, soluble, 3 binding protein
Snapc5	-1.0482	-2.0679	8.90E-08	small nuclear RNA activating complex, polypeptide 5
Tdg	-1.0644	-2.0912	1.42E-13	thymine DNA glycosylase
Hmga1	-1.0781	-2.1113	1.77E-14	high mobility group AT-hook 1
Pot1a	-1.0806	-2.1149	4.83E-06	protection of telomeres 1A
Mettl23	-1.1050	-2.1510	3.72E-15	methyltransferase like 23
Pop4	-1.1115	-2.1607	2.00E-14	processing of precursor 4, ribonuclease P/MRP family, (S. cerevisiae)
Rsad2	-1.1131	-2.1631	5.91E-15	radical S-adenosyl methionine domain containing 2
Col6a3	-1.1265	-2.1833	9.18E-24	collagen, type VI, alpha 3
Poln	-1.1605	-2.2353	1.14E-12	DNA polymerase N
Gm14005	-1.1702	-2.2504	9.71E-07	predicted gene 14005
Gm13091	-1.2002	-2.2977	2.63E-26	predicted gene 13091
Zfp758	-1.2032	-2.3025	6.16E-11	zinc finger protein 758
Cd44	-1.2213	-2.3315	1.09E-23	CD44 antigen
Nat8l	-1.2338	-2.3518	7.55E-23	N-acetyltransferase 8-like
AC126055.1	-1.2418	-2.3649	5.15E-09	Mus musculus BAC clone RP23-368O6 from chromosome 16
Pcdh17	-1.2911	-2.4471	7.85E-25	protocadherin 17
Ddx3y	-1.3000	-2.4622	3.17E-21	DEAD box helicase 3, Y-linked
Limch1	-1.3085	-2.4769	3.05E-25	LIM and calponin homology domains 1
Slc26a11	-1.3105	-2.4803	7.08E-11	solute carrier family 26, member 11
Pygm	-1.3447	-2.5398	1.28E-35	muscle glycogen phosphorylase
Galnt12	-1.5541	-2.9364	4.67E-27	polypeptide N-acetylgalactosaminyltransferase 12
Hgsnat	-1.5554	-2.9391	2.27E-37	heparan-alpha-glucosaminide N-acetyltransferase
Slc2a9	-1.5758	-2.9810	5.60E-12	solute carrier family 2 (facilitated glucose transporter), member 9
Emilin2	-1.6544	-3.1479	6.17E-30	elastin microfibril interfacer 2
Slc22a15	-1.7609	-3.3892	3.83E-17	solute carrier family 22 (organic anion/cation transporter), member 15
Gm26782	-1.7916	-3.4620	2.66E-12	predicted gene, 26782
Aox4	-1.8113	-3.5096	1.79E-20	aldehyde oxidase 4
Zfp365	-1.8248	-3.5426	6.34E-61	zinc finger protein 365
Avp	-1.9161	-3.7741	6.30E-40	arginine vasopressin
Lgsn	-2.2780	-4.8500	8.07E-87	lengsin, lens protein with glutamine synthetase domain
Atp6v0c	-3.0317	-8.1780	6.76E-55	ATPase, H+ transporting, lysosomal V0 subunit C
Sez6l	-3.0822	-8.4692	8.42E-35	seizure related 6 homolog like
Gm5345	-3.1245	-8.7209	2.05E-29	predicted gene 5345
H2-T24	-6.3309	-80.4999	1.89E-86	histocompatibility 2, T region locus 24
H2-K2	-10.4417	-1390.8174	1.55E-109	histocompatibility 2, K region locus 2

FC - fold change, FDR - false discovery rate

Table S4. Differentially regulated genes (FC > 2, FDR = 0.05) in the *Epha2* -indel722 lens (P7)

Gene_id	logFC.indel722.wt	FC.indel722.wt	FDR.indel722.wt	Description
**Upregulated**				
Tuba1c	6.4881	89.7658	0.00E+00	tubulin, alpha 1C
Rps3a3	6.1460	70.8180	9.84E-212	ribosomal protein S3A3
Rpsa-ps10	5.1763	36.1595	0.00E+00	ribosomal protein SA, pseudogene 10
Hmga1b	4.9157	30.1844	1.06E-65	high mobility group AT-hook 1B
Atp6v0c-ps2	4.5701	23.7539	2.49E-123	ATPase, H+ transporting, lysosomal V0 subunit C, pseudogene 2
Acer2	4.3154	19.9092	9.83E-245	alkaline ceramidase 2
Gm9825	4.1742	18.0529	3.76E-99	predicted gene 9825
Gm6969	4.0725	16.8240	1.15E-69	predicted pseudogene 6969
Draxin	3.5982	12.1104	2.50E-151	dorsal inhibitory axon guidance protein
Dcdc2a	2.5884	6.0145	4.74E-41	doublecortin domain containing 2a
Grik3	2.1785	4.5267	5.04E-50	glutamate receptor, ionotropic, kainate 3
Cabp5	2.1029	4.2957	1.11E-58	calcium binding protein 5
Wdfy1	2.0947	4.2714	2.81E-17	WD repeat and FYVE domain containing 1
Gm6472	2.0056	4.0156	8.56E-35	predicted pseudogene 6472
Gm14005	1.8656	3.6441	1.80E-33	predicted gene 14005
Rpl14-ps1	1.7897	3.4575	1.08E-42	ribosomal protein L14, pseudogene 1
Slc7a11	1.7843	3.4446	1.01E-57	solute carrier family 7 (cationic amino acid transporter, y+ system), member 11
Xist	1.7673	3.4042	3.07E-45	inactive X specific transcripts
Gm10382	1.6981	3.2447	4.90E-15	predicted gene 10382
Pcdhac2	1.6381	3.1127	2.68E-23	protocadherin alpha subfamily C, 2
Igf2	1.6313	3.0978	3.06E-35	insulin-like growth factor 2
Gm20075	1.5934	3.0176	1.89E-25	predicted gene 20075
Gm42808	1.5441	2.9161	1.27E-16	predicted gene 42808
Slc1a4	1.5436	2.9153	9.81E-43	solute carrier family 1 (glutamate/neutral amino acid transporter), member 4
Hmgcs2	1.5268	2.8814	7.55E-16	3-hydroxy-3-methylglutaryl-Coenzyme A synthase 2
Fmo1	1.5074	2.8430	4.22E-13	flavin containing monooxygenase 1
Col26a1	1.4884	2.8058	1.05E-10	collagen, type XXVI, alpha 1
Gstp2	1.4709	2.7720	1.91E-29	glutathione S-transferase, pi 2
Ece1	1.3953	2.6304	6.64E-38	endothelin converting enzyme 1
Bckdhb	1.3929	2.6260	2.95E-29	branched chain ketoacid dehydrogenase E1, beta polypeptide
Gstk1	1.3845	2.6109	1.18E-15	glutathione S-transferase kappa 1
Prkar1b	1.3756	2.5948	5.00E-25	protein kinase, cAMP dependent regulatory, type I beta
Mxra8	1.3011	2.4641	8.97E-22	matrix-remodelling associated 8
Dusp4	1.2791	2.4268	1.81E-19	dual specificity phosphatase 4
Pros1	1.2610	2.3965	3.57E-15	protein S (alpha)
Pde10a	1.2246	2.3370	2.96E-28	phosphodiesterase 10A
Gm47283	1.2166	2.3240	2.76E-10	predicted gene 47283
Gstp1	1.2040	2.3038	3.60E-30	glutathione S-transferase, pi 1
Mad2l2	1.2024	2.3013	2.27E-14	MAD2 mitotic arrest deficient-like 2
Npr1	1.1997	2.2968	7.37E-10	natriuretic peptide receptor 1
Fam135a	1.1316	2.1910	2.42E-14	family with sequence similarity 135, member A
Cst3	1.1304	2.1892	3.76E-26	cystatin C
Lrrc49	1.1197	2.1731	5.17E-17	leucine rich repeat containing 49
Gsta4	1.1070	2.1540	1.83E-08	glutathione S-transferase, alpha 4
D430041D05Rik	1.0852	2.1217	6.07E-21	RIKEN cDNA D430041D05 gene
Asph	1.0822	2.1173	5.37E-19	aspartate-beta-hydroxylase
Cenpf	1.0806	2.1150	7.59E-07	centromere protein F
Tgoln1	1.0801	2.1142	7.39E-17	trans-golgi network protein
Hspb8	1.0791	2.1127	1.95E-08	heat shock protein 8
Prkn	1.0757	2.1078	3.82E-09	parkin RBR E3 ubiquitin protein ligase
Ttyh1	1.0755	2.1074	3.39E-12	tweety family member 1
Cacna1a	1.0736	2.1047	3.32E-12	calcium channel, voltage-dependent, P/Q type, alpha 1A subunit
Ftx	1.0724	2.1029	4.03E-07	Ftx transcript, Xist regulator (non-protein coding)
Tent5a	1.0682	2.0968	4.48E-07	terminal nucleotidyltransferase 5A
Ctsl	1.0651	2.0923	4.44E-23	cathepsin L
9630028B13Rik	1.0511	2.0721	3.08E-07	RIKEN cDNA 9630028B13 gene
Shc3	1.0495	2.0698	1.43E-11	src homology 2 domain-containing transforming protein C3
Flywch1	1.0426	2.0599	4.40E-22	FLYWCH-type zinc finger 1
Ptgfr	1.0400	2.0562	5.00E-13	prostaglandin F receptor
Nes	1.0375	2.0526	5.58E-12	nestin
Nasp	1.0299	2.0419	3.70E-19	nuclear autoantigenic sperm protein (histone-binding)
Gpn2	1.0294	2.0412	2.47E-10	GPN-loop GTPase 2
Lamb2	1.0290	2.0406	9.45E-13	laminin, beta 2
Ntan1	1.0189	2.0263	1.79E-05	N-terminal Asn amidase
Kcnq1ot1	1.0121	2.0169	1.79E-06	KCNQ1 overlapping transcript 1
Cdk20	1.0076	2.0106	5.81E-08	cyclin-dependent kinase 20
Fbn1	1.0071	2.0098	1.13E-08	fibrillin 1
Bag3	1.0063	2.0088	9.98E-16	BCL2-associated athanogene 3
**Downregulated**				
Paqr7	-1.0066	-2.0092	1.49E-15	progestin and adipoQ receptor family member VII
Ankrd34c	-1.0169	-2.0236	3.73E-09	ankyrin repeat domain 34C
Pygl	-1.0213	-2.0297	8.58E-20	liver glycogen phosphorylase
Unc5b	-1.0347	-2.0487	5.60E-08	unc-5 netrin receptor B
Rgs5	-1.0699	-2.0993	5.48E-06	regulator of G-protein signaling 5
Anks6	-1.0832	-2.1188	8.59E-07	ankyrin repeat and sterile alpha motif domain containing 6
Megf11	-1.0866	-2.1237	2.46E-10	multiple EGF-like-domains 11
Kcnq4	-1.1210	-2.1749	3.68E-09	potassium voltage-gated channel, subfamily Q, member 4
Mlh1	-1.1349	-2.1960	1.54E-10	mutL homolog 1
Snapc5	-1.1647	-2.2419	1.17E-09	small nuclear RNA activating complex, polypeptide 5
Hmgn2	-1.1670	-2.2454	5.22E-22	high mobility group nucleosomal binding domain 2
Pop4	-1.1687	-2.2480	3.12E-16	processing of precursor 4, ribonuclease P/MRP family, (S. cerevisiae)
Bfsp2	-1.1808	-2.2670	9.75E-30	beaded filament structural protein 2, phakinin
Irak1bp1	-1.2523	-2.3822	1.69E-07	interleukin-1 receptor-associated kinase 1 binding protein 1
Epha2	-1.2887	-2.4430	3.24E-29	Eph receptor A2
Dhcr7	-1.3317	-2.5170	5.22E-31	7-dehydrocholesterol reductase
Tbc1d30	-1.3552	-2.5584	4.84E-18	TBC1 domain family, member 30
Csmd2	-1.3927	-2.6257	2.77E-17	CUB and Sushi multiple domains 2
Cited4	-1.4102	-2.6578	3.78E-12	Cbp/p300-interacting transactivator, with Glu/Asp-rich carboxy-terminal domain, 4
Rab3b	-1.4556	-2.7427	7.81E-26	RAB3B, member RAS oncogene family
Cacna1h	-1.4668	-2.7641	3.43E-11	calcium channel, voltage-dependent, T type, alpha 1H subunit
Galnt12	-1.5111	-2.8503	3.95E-26	polypeptide N-acetylgalactosaminyltransferase 12
E530011L22Rik	-1.5452	-2.9185	2.41E-16	RIKEN cDNA E530011L22 gene
Akr1e1	-1.5622	-2.9530	2.69E-26	aldo-keto reductase family 1, member E1
Sulf2	-1.6155	-3.0642	3.13E-22	sulfatase 2
C920006O11Rik	-1.6166	-3.0665	9.93E-17	RIKEN cDNA C920006O11 gene
Fhad1	-1.7357	-3.3304	1.23E-13	forkhead-associated (FHA) phosphopeptide binding domain 1
Avp	-1.8520	-3.6099	2.07E-38	arginine vasopressin
Efhd2	-1.8815	-3.6845	1.58E-65	EF hand domain containing 2
Zfp758	-1.9240	-3.7948	3.92E-24	zinc finger protein 758
Lgals3bp	-2.0180	-4.0502	5.90E-23	lectin, galactoside-binding, soluble, 3 binding protein
Rsad2	-2.1182	-4.3415	8.35E-46	radical S-adenosyl methionine domain containing 2
Rab6b	-2.3046	-4.9404	9.63E-50	RAB6B, member RAS oncogene family
Pttg1	-2.5100	-5.6961	1.23E-35	pituitary tumor-transforming gene 1
Camk2n1	-2.8513	-7.2163	4.77E-19	calcium/calmodulin-dependent protein kinase II inhibitor 1
Gm13091	-2.9814	-7.8975	2.32E-130	predicted gene 13091
Atp6v0c	-3.3342	-10.0854	5.40E-63	ATPase, H+ transporting, lysosomal V0 subunit C
Nipal3	-4.7315	-26.5666	4.42E-180	NIPA-like domain containing 3
H2-T24	-5.3268	-40.1345	1.09E-81	histocompatibility 2, T region locus 24
Lgsn	-7.1733	-144.3408	0.00E+00	lengsin, lens protein with glutamine synthetase domain
Lyzl4	-9.3364	-646.4345	6.75E-58	lysozyme-like 4
Clic5	-10.6303	-1584.9850	0.00E+00	chloride intracellular channel 5

FC - fold change, FDR - false discovery rate

Table S5. Differentially regulated genes (FC > 2, FDR = 0.05) in the *Epha2* -null lens (P7)

Gene_id	logFC.null.wt	FC.null.WT	FDR.null.wt	Description
**Upregulated**				
Eif3j2	3.8509	14.4292	1.33E-72	eukaryotic translation initiation factor 3, subunit J2
Wdfy1	3.4059	10.5994	1.22E-59	WD repeat and FYVE domain containing 1
Stard9	2.8627	7.2737	5.36E-29	START domain containing 9
Dcdc2a	2.6423	6.2432	1.06E-41	doublecortin domain containing 2a
Gm47283	2.1902	4.5638	3.84E-38	predicted gene 47283
Gm10382	2.0316	4.0886	8.25E-22	predicted gene 10382
Hmgcs2	1.8441	3.5904	1.29E-23	3-hydroxy-3-methylglutaryl-Coenzyme A synthase 2
Xist	1.8268	3.5474	4.24E-47	inactive X specific transcripts
Plk5	1.7507	3.3652	9.36E-32	polo like kinase 5
Cabp5	1.7392	3.3385	3.14E-38	calcium binding protein 5
Gstp2	1.7377	3.3350	5.23E-41	glutathione S-transferase, pi 2
Camk2n1	1.6816	3.2078	8.30E-21	calcium/calmodulin-dependent protein kinase II inhibitor 1
Gm42808	1.6371	3.1105	1.98E-18	predicted gene 42808
Gm14005	1.5430	2.9140	4.69E-21	predicted gene 14005
Anxa3	1.5270	2.8818	4.03E-31	annexin A3
Gcg	1.5173	2.8626	1.05E-43	glucagon
Prkar1b	1.5103	2.8487	9.10E-30	protein kinase, cAMP dependent regulatory, type I beta
Cd59a	1.5087	2.8456	7.39E-24	CD59a antigen
Stambpl1	1.5087	2.8456	3.85E-43	STAM binding protein like 1
Map1a	1.4910	2.8109	1.26E-31	microtubule-associated protein 1 A
Tent5a	1.4869	2.8029	3.49E-14	terminal nucleotidyltransferase 5A
Dusp4	1.4351	2.7040	3.34E-24	dual specificity phosphatase 4
Col26a1	1.3996	2.6383	8.45E-09	collagen, type XXVI, alpha 1
Gstp1	1.3626	2.5715	3.26E-38	glutathione S-transferase, pi 1
Myo10	1.3568	2.5611	7.14E-16	myosin X
Ano1	1.3541	2.5564	1.39E-22	anoctamin 1, calcium activated chloride channel
Gm13883	1.3455	2.5412	1.86E-18	predicted gene 13883
Gstk1	1.3409	2.5331	7.44E-14	glutathione S-transferase kappa 1
Sox6	1.3375	2.5271	8.16E-11	SRY (sex determining region Y)-box 6
Npr1	1.3301	2.5142	5.01E-12	natriuretic peptide receptor 1
Kctd12	1.3081	2.4761	3.66E-19	potassium channel tetramerisation domain containing 12
Emilin1	1.2817	2.4312	5.88E-29	elastin microfibril interfacer 2
Hectd1	1.2633	2.4005	9.48E-33	HECT domain E3 ubiquitin protein ligase 1
Acta2	1.2425	2.3661	9.07E-30	actin, alpha 2, smooth muscle, aorta
Anxa5	1.2223	2.3332	7.28E-20	annexin A5
Sorcs2	1.1971	2.2928	3.30E-13	sortilin-related VPS10 domain containing receptor 2
Tnxb	1.1961	2.2911	2.14E-14	tenascin XB
Sqstm1	1.1825	2.2697	1.62E-28	sequestosome 1
Ptgfr	1.1709	2.2515	3.17E-16	prostaglandin F receptor
9630028B13Rik	1.1586	2.2323	2.29E-08	RIKEN cDNA 9630028B13 gene
Ano4	1.1450	2.2115	8.53E-15	anoctamin 4
Lgals1	1.1400	2.2038	6.28E-22	lectin, galactose binding, soluble 1
Rasgrp1	1.1396	2.2033	9.29E-16	RAS guanyl releasing protein 1
Ttyh1	1.1330	2.1931	3.45E-13	tweety family member 1
Mtus2	1.1276	2.1850	8.88E-08	mitochondrial tumor suppressor 1
Jag2	1.1202	2.1737	5.78E-08	jagged 2
Rgs8	1.1167	2.1685	2.45E-22	regulator of G-protein signaling 8
Pcdhac2	1.1144	2.1650	1.78E-09	protocadherin alpha subfamily C, 2
Shc3	1.1008	2.1447	1.92E-12	src homology 2 domain-containing transforming protein C3
Bc1	1.0901	2.1289	1.99E-23	brain cytoplasmic RNA 1
Cacna1a	1.0799	2.1139	4.70E-12	calcium channel, voltage-dependent, P/Q type, alpha 1A subunit
Cacng4	1.0798	2.1137	1.04E-06	calcium channel, voltage-dependent, gamma subunit 4
Robo1	1.0780	2.1112	2.31E-13	roundabout guidance receptor 1
Necap2	1.0757	2.1077	6.48E-22	NECAP endocytosis associated 2
Kcnq1ot1	1.0745	2.1060	5.02E-07	KCNQ1 overlapping transcript 1
Fbn1	1.0715	2.1016	1.87E-09	fibrillin 1
Galnt12	1.0695	2.0988	1.02E-17	polypeptide N-acetylgalactosaminyltransferase 12
Caprin2	1.0652	2.0925	4.08E-24	caprin family member 2
Gale	1.0640	2.0907	4.50E-09	galactose-4-epimerase, UDP
Bag3	1.0470	2.0662	1.03E-16	BCL2-associated athanogene 3
Draxin	1.0410	2.0576	1.92E-09	dorsal inhibitory axon guidance protein
Vash2	1.0326	2.0458	8.11E-10	vasohibin 2
Hmcn1	1.0298	2.0418	8.04E-08	hemicentin 1
Hspb8	1.0207	2.0290	3.55E-07	heat shock protein 8
Osbpl6	1.0186	2.0260	1.95E-10	oxysterol binding protein-like 3
Trp53inp1	1.0177	2.0247	1.80E-06	transformation related protein 53 inducible nuclear protein 1
Ntan1	1.0116	2.0161	3.17E-05	N-terminal Asn amidase
S100a10	1.0083	2.0115	8.25E-13	S100 calcium binding protein A10 (calpactin)
**Downregulated**				
Actn2	-1.0134	-2.0186	4.63E-18	actinin alpha 2
Sulf2	-1.0526	-2.0743	4.72E-10	sulfatase 2
Insm1	-1.0698	-2.0991	2.93E-04	insulinoma-associated 1
Dhcr7	-1.1237	-2.1790	8.42E-22	7-dehydrocholesterol reductase
Iqsec3	-1.1461	-2.2131	3.55E-07	IQ motif and Sec7 domain 3
Cx3cl1	-1.1827	-2.2699	7.63E-18	chemokine (C-X3-C motif) ligand 1
Gm31036	-1.2254	-2.3383	2.92E-12	predicted gene 31036
Clic5	-1.2266	-2.3402	5.88E-29	chloride intracellular channel 5
Gadd45a	-1.2343	-2.3526	9.31E-17	growth arrest and DNA-damage-inducible 45 alpha
Dbf4	-1.2382	-2.3590	3.30E-13	DBF4 zinc finger
Ngef	-1.2916	-2.4481	1.32E-30	neuronal guanine nucleotide exchange factor
Rsad2	-1.4839	-2.7971	2.83E-24	radical S-adenosyl methionine domain containing 2
Chrna4	-1.5545	-2.9373	8.97E-29	cholinergic receptor, nicotinic, alpha polypeptide 4
Gm13091	-1.5692	-2.9675	2.23E-43	predicted gene 13091
Unc5b	-1.6447	-3.1269	5.67E-16	unc-5 netrin receptor B
Tbc1d30	-1.6746	-3.1924	3.55E-24	TBC1 domain family, member 30
Lgsn	-1.6815	-3.2077	2.09E-49	lengsin, lens protein with glutamine synthetase domain
Gpd1l	-1.8983	-3.7278	4.72E-67	glycerol-3-phosphate dehydrogenase 1-like
Avp	-2.7293	-6.6315	2.62E-64	arginine vasopressin
Sox2ot	-2.8801	-7.3619	6.39E-123	SOX2 overlapping transcript (non-protein coding)
AI506816	-5.1627	-35.8195	9.66E-236	expressed sequence AI506816

FC - fold change, FDR - false discovery rate
